# Emerging trends of fluorescence lifetime imaging microscopy (FLIM): advances, challenges, and prospects

**DOI:** 10.52601/bpr.2025.250024

**Published:** 2026-08-31

**Authors:** Baoyi Wang, Meilin Li, Xiaoshuai Huang, Bei Liu

**Affiliations:** 1 National Biomedical Imaging Center, College of Future Technology, Peking University, Beijing 100871, China; 2 Key laboratory of Carcinogenesis and Translational Research (Ministry of Education/Beijing), Peking University Cancer Hospital & Institute, Beijing 100142, China; 3 Beijing Laboratory of Biomedical Imaging, Beijing 100871, China

**Keywords:** FLIM, Phasor analysis, Multiplexing, Biosensor, Deep learning, Super-resolution microscopy

## Abstract

This mini-review provides a concise overview of the latest advances in fluorescence lifetime imaging microscopy (FLIM). It discusses both time-domain and frequency-domain techniques, analysis methods − including phasor approaches and deep learning, and highlights applications in multiplexed imaging and quantitative biosensing. Furthermore, FLIM-empowered multimodal imaging approaches aimed at enhancing spatial and temporal resolution are discussed. Persistent challenges, including photon efficiency, probe sensitivity, and achieving high-speed imaging in live-cell environments, are critically assessed, outlining pathways toward future innovations.

## INTRODUCTION

Fluorescence imaging has become an indispensable means to investigate cellular structures and functions. Conventional approaches employ spectrum-resolved intensity or wavelength-ratiometric measurements to simultaneously investigate multiple targets. However, quantitation dependent on fluorescence intensity signals is influenced by spectral crosstalk, sample concentration, light source intensity, and probe photobleaching (Lippincott-Schwartz and Patterson 2003).

Fluorescence decay, commonly characterized by the fluorescence lifetime, is a unique property that serves as a “fingerprint” for the excited-state dynamics of fluorophores (Boens *et al.* 2007), carrying the biophysical and biochemical properties of the surrounding environment. Fluorescence Lifetime Imaging Microscopy (FLIM), first introduced in the late 1980s (Bugiel *et al.* 1989; König 2018; Schneckenburger 1985; Schneckenburger *et al.* 1987), has become a routine tool in biological study. FLIM is widely applied for studying inter-molecular interactions (Bücherl *et al.* 2010; Jares-Erijman and Jovin 2003; Margineanu *et al.* 2016), protein conformations (Calleja *et al.* 2003; Eggan *et al.* 2024), analyte concentrations (Rennick *et al.* 2022; Simonyan *et al.* 2024), metabolic states (Blacker *et al.* 2014; Lakowicz *et al.* 1992), cell cycle dynamic (Shirmanova *et al.* 2021; Tan *et al.* 2024), and lesion detection (Dysli *et al.* 2017; König *et al.* 1999). Based on the strategy used to determine fluorescence decay properties, FLIM is primarily classified into two categories: time domain (tdFLIM) and frequency domain (fdFLIM). The time domain method is characterized by precisely determining the arrival time of emitted photons upon excitation of a pulsed laser source, allowing for the statistical derivation of fluorescence decay curves (Lakowicz 2006). In contrast, the frequency domain method measures the phase shift and demodulation between the fluorescence emission waveform and the periodic laser excitation waveform (Becker 2012).

TdFLIM is typically combined with the raster scanning of a confocal system with pixel-wise time-correlated single photon counting (TCSPC) (Torrado *et al.* 2024). TdFLIM provides superior contrast, high sensitivity, and the ability to perform label-free imaging. Consequently, tdFLIM rapidly demonstrated its clinical potential in the 1990s (Koenig and Schneckenburger 1994; König *et al.* 1999) and fundamental biological studies (Bacia *et al*. 2006; Becker *et al*. 2001). In the past decade, the advent of phasor analysis, deep learning, and novel probes has rapidly expanded tdFLIM’s multiplexing capabilities within the same spectral window (Niehörster *et al.* 2016; Starling *et al.* 2023).

On the other hand, fdFLIM is more qualified for high-speed wide-field imaging (Becker 2012). The apparatus for measuring the fluorescence phase shift was described in the 1960s (Bailey and Rollefson 1953). After stable variable-frequency instruments emerged in the mid-1980s, fdFLIM has enabled lifetime unmixing of multiple fluorophores (Bright *et al.* 1990) and integrates effectively with advanced imaging methods such as two-photon microscopy (Li *et al.* 2025), electro-optic microscopy (Bowman *et al.* 2023), and high-content screening (Kanno *et al.* 2024).

This review covers the fundamental principles of tdFLIM and fdFLIM, commonly employed data analysis strategies, with highlights of recent advances in multiplexed imaging and biosensing.

## FUNDAMENTAL PRINCIPLE

### Time domain

**Figure 1:**
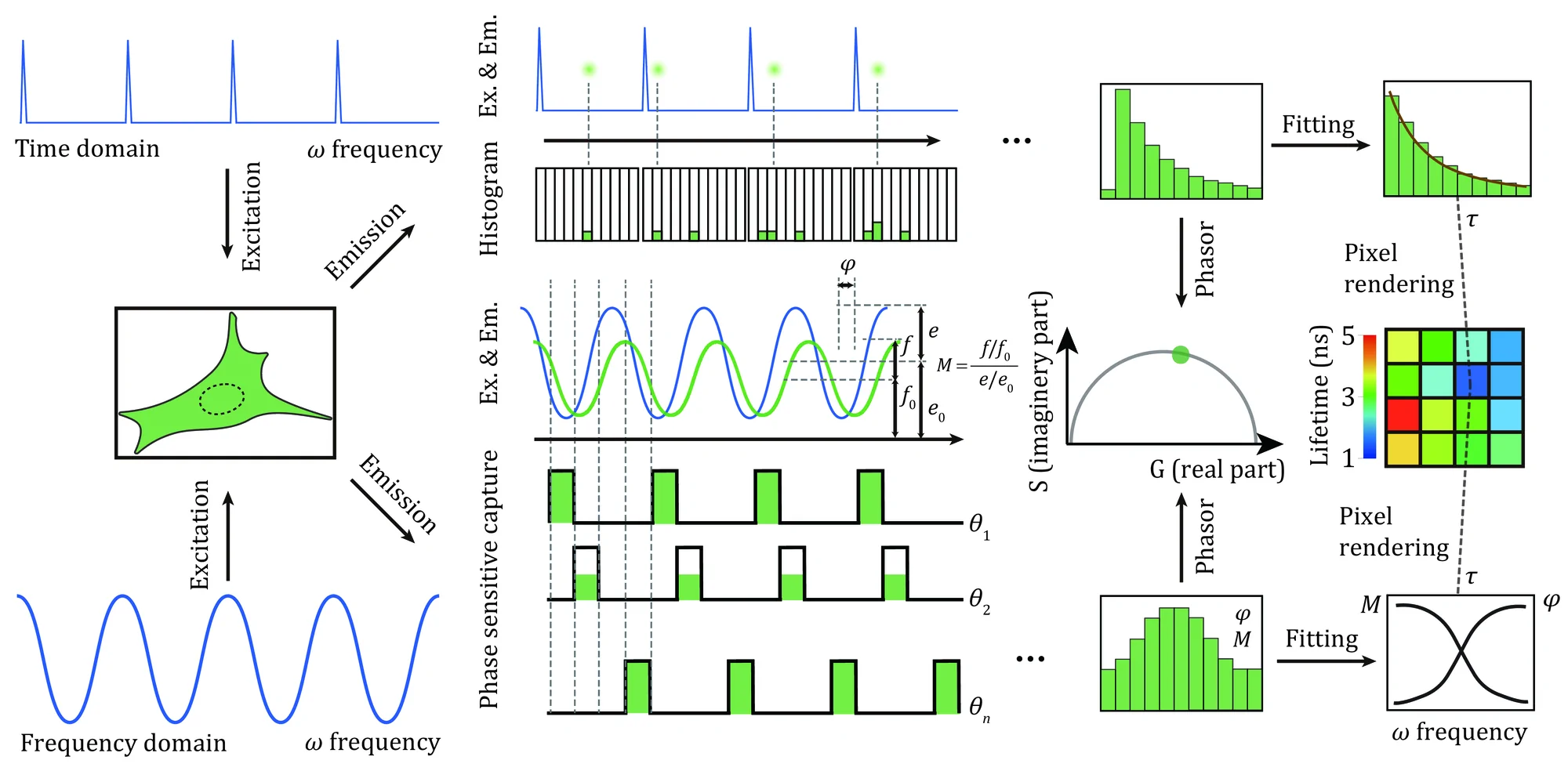
FLIM principles. Schematic overview of fluorescence lifetime data acquisition and analysis in time−domain (td) and frequency−domain (fd) modes. In tdFLIM, photon arrival times are recorded after each pulsed excitation to build a decay histogram, which can be analyzed by multi-exponential curve fitting or transformed into the phasor space for model-free lifetime extraction. In fdFLIM, the emission is measured under high-frequency intensity modulation, yielding phase shift and demodulation factors that can likewise be processed by fitting or phasor analysis. Both approaches ultimately render lifetime maps as pseudocolor images for biological interpretation

The most used tdFLIM strategy is the time-correlated single photon counting (TCSPC) combined with scanning confocal microscopy. The sample is excited by raster scanning a mode-locked pulsed laser with a high repetition frequency, ensuring that in each cycle, the signals detected by the photomultiplier tube originate from a single photon (Fig. 1, upper left panel). The time difference between each detected photon and the excitation pulse is recorded as a timestamp for that photon. After collecting a substantial number of photons, a histogram of photon counts versus timestamps is generated (Fig. 1, upper right panel). The trailing edge of this histogram characterizes the fluorescence decay curve. By fitting the total intensity $ {F}_{\delta }\left(t\right) $ over time with multi-exponential model (Lakowicz 2006):

1 $ {F}_{\delta }\left(t\right)={\sum }_{s}{a}_{s}{e}^{-t/{\tau }_{s}} , $

where $ {F}_{\delta } $ is the fluorescence intensity over time, and the impulse response function of a fluorescence lifetime imaging system. $ {a}_{s} $ is the pre-exponential factor and $ {\tau }_{s} $ is the decay time of component * s *. Notably, the recovered values of $ {a}_{s} $ and $ {\tau }_{s} $ from the model do not necessarily have physical meaning. They are just projections in the mathematical sense in exponential functional space (Lakowicz 2006).

### Frequency domain

FdFLIM is mostly based on the phase shift method (Bailey and Rollefson 1953). The lifetimes of fluorescence are determined by the phase difference and demodulation between the high-frequency intensity-modulated excitation light and the emitted fluorescence (Fig. 1, lower panel). The emission fluorescence signal is the result of the convolution of the excitation light and the impulse response, which is also characterized by the multi-exponential model:

2 $ F\left(t\right)={\int }_{0}^{t}E\left({t}'\right){F}_{\delta }\left(t-{t}'\right)dt'={F}_{0}\left(2{m}_{\omega }\mathrm{cos}\left(\omega t+{\varphi }_{\omega }\right)+1\right) , $

where $ E\left(t\right) $ is the expression of periodic excitation light at a frequency of * ω ,* $ F\left(t\right) $ is the expression of the emission fluorescence signal, $ {F}_{0} $ is the mean fluorescent intensity over time, and phase shift $ {\varphi }_{\omega } $ and demodulation $ {m}_{\omega } $ are the differences between the emitted signal and the excitation signal. The cosine coefficients $ {G}_{\omega } $ and sine coefficients $ {S}_{\omega } $ in the trigonometric Fourier series of the emission signal are characterized as Eqs. 3 and 4 (Lakowicz 2006).

3 $ {G}_{\omega }={\sum }_{s}\frac{{a}_{s}{\tau }_{s}}{1+{\left(\omega {\tau }_{s}\right)}^{2}}/{\sum }_{s}{a}_{s}{\tau }_{s} , $

4 $ {S}_{\omega }={\sum }_{s}\frac{{a}_{s}\omega {\left({\tau }_{s}\right)}^{2}}{1+{\left(\omega {\tau }_{s}\right)}^{2}}/{\sum }_{s}{a}_{s}{\tau }_{s} . $

The phase shift $ {\varphi }_{\omega } $ and demodulation $ {m}_{\omega } $ are related to the decay times $ {\tau }_{s} $ (Lakowicz 2006):

5 $ \mathrm{tan}{\varphi }_{\omega }={S}_{\omega }/{G}_{\omega } , $

6 $ {m}_{\omega }=\sqrt{{{S}_{\omega }}^{2}+{{G}_{\omega }}^{2}} . $

As frequency * ω * increases the phase shift increases from 0 to 90°, and the demodulation decreases from 1 to 0. Therefore, moderate modulation frequency should be chosen in order to produce a significant phase shift and intensity fluctuation signal simultaneously. A single modulation frequency can only determine the averaged time decay. Multiple fluorescence lifetime components can be decoupled by fitting the curves of phase and demodulation as functions of ample modulation frequencies (Lakowicz 2006) (Fig. 1, lower right panel).

The emission signal is acquired by gain-adjustable photoelectric sensors. The gain curve is a repetitive waveform with determinable phase shift at a homogeneous or slightly heterogeneous fundamental frequency with that of the excitation light (Fig. 1, lower middle panel). To balance signal strength and the errors between discrete summation and continuous integration, it is recommended that signals be acquired in 12 consecutive gain phase shifts. (Elder *et al.* 2006; Klarenbeek *et al.* 2015; Mukherjee *et al.* 2024; Raspe *et al.* 2016). To improve the acquisition speed and minimize artifacts, special phase-sensitive detection that simultaneously records two 180°-phase-shifted images is developed (Raspe *et al.* 2016).

## ANALYSIS TOOLS

### Phasor approach

Conventional approaches for tdFLIM and fdFLIM data fitting are based on non-linear least square regression, which is time-consuming and dependent on the choice of fitting models (Adhikari *et al.* 2023; Digman *et al.* 2008; Pelet *et al.* 2004). In contrast, the phasor method offers a fast and model-free alternative to exponential fitting, especially when dealing with multi-exponential decays and low photon counts (<100 photons) (Digman *et al*. 2008; Héliot and Leray 2021). The axes of the phasor plot represent the cosine coefficient $ {G}_{\omega } $ and sine coefficient $ {S}_{\omega } $ of the fundamental frequency term in the triangle series expansion of the fluorescent signal. In tdFLIM, they are derived from Eqs. 7 and 8.

7 $ {G}_{\omega }=\frac{\displaystyle\int _{0}^{T}I\left(t\right)\cdot \mathrm{cos}\left(\omega t\right)dt}{\displaystyle\int _{0}^{T}I\left(t\right)dt} , $

8 $ {S}_{\omega }=\frac{\displaystyle\int _{0}^{T}I\left(t\right)\cdot \mathrm{sin}\left(\omega t\right)dt}{\displaystyle\int _{0}^{T}I\left(t\right)dt} . $

In fdFLIM, they are calculated by Eqs. 9 and 10.

9 $ {G}_{\omega }={m}_{\omega }cos{\varphi }_{\omega } , $

10 $ {S}_{\omega }={m}_{\omega }sin{\varphi }_{\omega } . $

The universal semicircle rules of the phasor approach enable quick discrimination of anomalous signals and determination of multiplex fluorescence lifetimes (Redford and Clegg 2005).

### Deep learning-driven FLIM analysis

Due to the limited availability of training datasets for fluorescence lifetime imaging microscopy (FLIM), many deep neural networks (DNN) approaches utilize synthetic data, such as decay-modified MNIST images, to improve processing efficiency (Lin *et al.* 2025; Mannam *et al.* 2020; Smith *et al.* 2019; Yao *et al.* 2019). Most models focus on accelerating data fitting or enhancing the phasor approach for visualization (Héliot and Leray 2021; Lin *et al*. 2025; Smith *et al*. 2019; Zickus *et al*. 2020). However, decay-modified MNIST images are far too simplistic to capture the complex spatial heterogeneity, multi-exponential decay profiles, and noise distributions of real FLIM data. Furthermore, advanced models employing sparse photon sampling and spatial reconstruction via DL have been developed to enable faster FLIM imaging by integrating intensity information and effective noise reduction, using paired datasets of low- and high-quality FLIM images (Kapitany *et al.* 2024; Kapsiani *et al.* 2025; Shen *et al.* 2024; Xiao *et al.* 2023). However, the paired FLIM dataset remains very limited: SparseFLIM’s demo_test archive is under 100 MB (fewer than 100 paired 512 × 512 FLIM stacks) (Shen *et al.* 2024), whereas specialized spatial-resolution benchmarks such as BioSR comprise over 2200 low-resolution–high-resolution image pairs covering multiple organelles (CCPs, ER, MTs, F-actin) at 512 × 512 px (Qiao *et al.* 2021), and BioSR+ extends this collection to five structures (including Myosin-IIA) with eight signal levels per ROI (3.7 TB total) (Qiao 2022), underscoring the urgent need for similarly large, high-quality, organelle-annotated FLIM spatiotemporal datasets. Future directions for deep learning-driven FLIM are explored in the Section of Outlook.

### Computation platforms

Commercial software for FLIM analysis is generally stable and user-friendly; however, these solutions tend to be costly and lack the flexibility to meet all challenges (Torrado *et al.* 2024). In contrast, open-source FLIM analysis software has flourished in recent years, offering broad support for various FLIM data formats (Bernardi and Cardarelli 2023; Gao *et al*. 2020; Gottlieb *et al*. 2023; Schrimpf *et al*. 2018; Tan *et al*. 2024). Moreover, integration with platforms such as Napari (Wetzker *et al.* 2025) or FIJI (Gao *et al.* 2020; Schindelin *et al.* 2012) facilitates seamless downstream signal analysis.

## MULTIPLEXING AND BIOSENSING

### Multiplexing

Multiplexing via FLIM leverages variations in fluorescence lifetimes to label and differentiate between distinct cellular structures or functional molecules, providing a new coding perspective orthogonal to the fluorescence wavelength. However, unlike spectral imaging — where physical filters separate signals — lifetime-based multiplexing relies on post-acquisition algorithmic processing (Scipioni *et al.* 2021).

**Figure 2:**
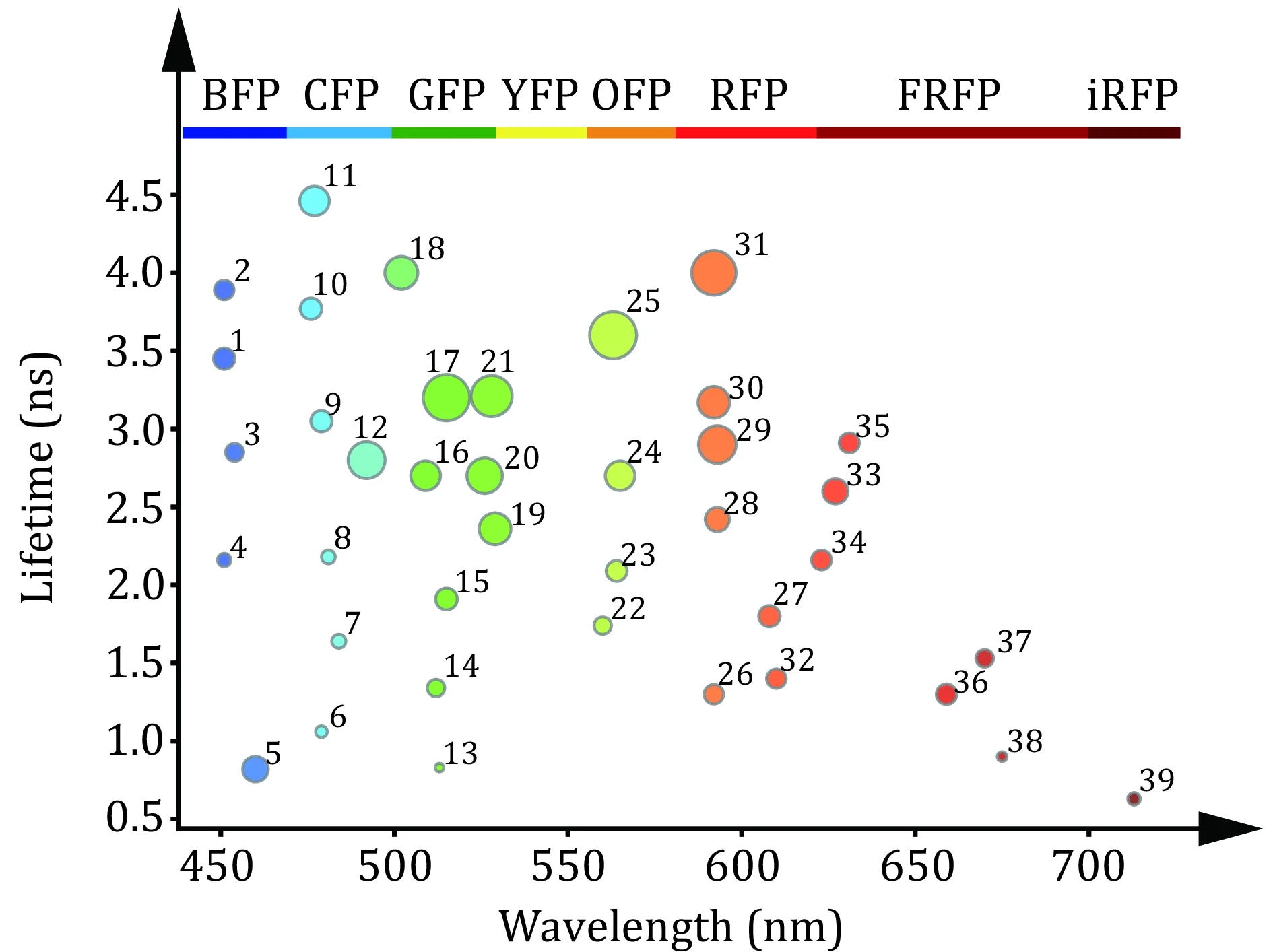
Scatter plot of example fluorescent proteins spanning the visible spectrum. Each circle’s position reflects the emission maximum and fluorescence lifetime, while the circle’s radius indicates the relative brightness of the FP, illustrating the diversity of probes available for multiplexed FLIM applications

Beyond advancements in algorithms, a critical factor in multiplexing lies in the development of probes with a narrow distribution of stable fluorescence lifetimes (Berezin and Achilefu 2010). Fluorescence labeling strategies include genetically encoded fluorescent proteins (FPs) (Tan *et al.* 2024), dyes (Mehl *et al.* 2024), and self-labeling enzymes (Frei *et al.* 2022). Figure 2 displays a scatter plot summarizing the recorded fluorescence lifetimes of various fluorescent proteins across different spectral ranges. The provenance and key photophysical parameters (emission maximum, measured lifetime, molecular brightness, category, and DOI reference) for each fluorescent protein depicted in Fig. 2 are compiled in Table 1. It is important to note that these lifetime values were obtained from different laboratories under varying conditions; hence, they should be regarded as relative estimates for arbitrary comparison rather than as absolute standards.

**Table 1:** FPs with different lifetimes across the spectrum

Order	Name	Emmision (nm)	Lifetime (ns)	Brightness	Category
1	EBFP2 (Tan *et al.* 2024)	451	3.45	17.9	BFP
2	EBFP2-T62V (Tan *et al.* 2024)	451	3.89	15	BFP
3	EBFP2-H148A (Tan *et al.* 2024)	454	2.85	12.9	BFP
4	EBFP-E222D (Tan *et al.* 2024)	451	2.16	7	BFP
5	BruSLEE (Mamontova *et al.* 2018)	460	0.82	25.8	BFP
6	mTurquoise2-T203G (Tan *et al.* 2024)	479	1.06	5.3	CFP
7	mTurquoise2-T203L (Tan *et al.* 2024)	484	1.64	7.8	CFP
8	mTurquoise2-S205A(Tan *et al.* 2024)	481	2.18	7.5	CFP
9	mTurquoise2-F146Q (Tan *et al.* 2024)	479	3.05	17.9	CFP
10	mTurquoise2-V68C (Tan *et al.* 2024)	476	3.77	18.4	CFP
11	mTurquoise2-T62C (Tan *et al.* 2024)	477	4.46	34.6	CFP
12	mTFP1 (Starling *et al.* 2023)	492	2.8	54	CFP
13	mNeonGreen-R195A (Tan *et al.* 2024)	513	0.83	2.8	GFP
14	mNeonGreen-F155L (Tan *et al.* 2024)	512	1.34	12	GFP
15	mNeonGreen-W157G (Tan *et al.* 2024)	515	1.91	18.6	GFP
16	EGFP (Starling *et al.* 2023)	509	2.7	35	GFP
17	mClover3 (Starling *et al.* 2023)	515	3.2	85	GFP
18	NowGFP (Starling *et al.* 2023)	502	4	43	GFP
19	mVenus-S205A (Tan *et al.* 2024)	529	2.36	40	YFP
20	mVenus-H148V (Tan *et al.* 2024)	526	2.7	50	YFP
21	mVenus (Tan *et al.* 2024)	528	3.21	66.6	YFP
22	mOrange-M163A (Tan *et al.* 2024)	560	1.74	11.8	OFP
23	mOrange-S146N (Tan *et al.* 2024)	564	2.09	17.2	OFP
24	mOrange2 (Tan *et al.* 2024)	565	2.7	34.8	OFP
25	mOrange-I161L (Tan *et al.* 2024)	563	3.6	87.2	OFP
26	mScarlet-H (Starling *et al.* 2023)	592	1.3	14.8	RFP
27	Fusionred (Manna *et al.* 2018)	608	1.8	18	RFP
28	mScarlet-M164L (Tan *et al.* 2024)	593	2.42	23.1	RFP
29	mScarlet-I (Starling *et al.* 2023)	593	2.9	56.1	RFP
30	mScarlet-F178A (Tan *et al.* 2024)	592	3.17	39.9	RFP
31	mScarlet3 (Gadella *et al.* 2023)	592	4	78	RFP
32	mCherry (Starling *et al.* 2023)	610	1.4	15	RFP
33	mKate2 (Tan *et al.* 2024)	627	2.6	25	FRFP
34	mKate2-S144T (Tan *et al.* 2024)	623	2.16	15.5	FRFP
35	mKate2-T61Q (Tan *et al.* 2024)	631	2.91	16	FRFP
36	mCardinal (Canty *et al.* 2018)	659	1.3	16.5	FRFP
37	emiRFP670 (Zhang *et al.* 2023a)	670	1.53	12.24	FRFP
38	TagRFP675 (Canty *et al.* 2018)	675	0.9	3.7	FRFP
39	iRFP713 (Canty *et al.* 2018)	713	0.63	6.1	iRFP

#### Genetically encoded fluorescent proteins (FPs)

The fluorescence lifetime of green fluorescent proteins (GFPs) typically ranges from 2.3 to 3.5 ns (Mamontova *et al.* 2018). However, lifetimes can vary with pH, temperature, environmental conditions, instrumentation, and analysis methods. Consequently, comparing absolute lifetimes across different laboratories is challenging. Recent advancements have produced engineered FPs with a wider range of lifetimes across diverse spectral windows (Aoyama *et al.* 2023; Bindels *et al.* 2017; Gadella *et al.* 2023; Mamontova *et al.* 2018; Mukherjee *et al.* 2022; Tan *et al.* 2024), as illustrated in Fig. 2. For instance, a study from Westlake University successfully distinguished nine intracellular structures using a family of FPs (Tan *et al.* 2024).

#### Dyes & Self-labeling enzymes

*In vitro*, some dyes exhibit lifetimes as long as 10 ns (Berezin and Achilefu 2010); however, *in vivo*, their lifetimes are generally comparable to those of FPs (Frei *et al.* 2022; Vallmitjana *et al.* 2020). Self-labeling enzymes, such as engineered FAST tags (Bogdanova *et al.* 2024; El Hajji *et al.* 2024), and HaloTag variants (Frei *et al.* 2022) have been effectively used for multiplexing within a single spectral channel, with a few attempts towards resolving three- or four-target separation even under STED conditions (Gonzalez Pisfil *et al.* 2022; Tan *et al.* 2024; Wang *et al.* 2025).

### Quantitative biosensing

When the lifetime of a fluorescent probe exhibits a robust correlation with the physicochemical properties of the target molecule or biological microenvironment, it can be utilized as a biosensor for the quantitative characterization of biological events. Unlike the absolute quantification of lifetimes in multiplexing, biosensing focuses on the relative change of the lifetimes.

**Figure 3:**
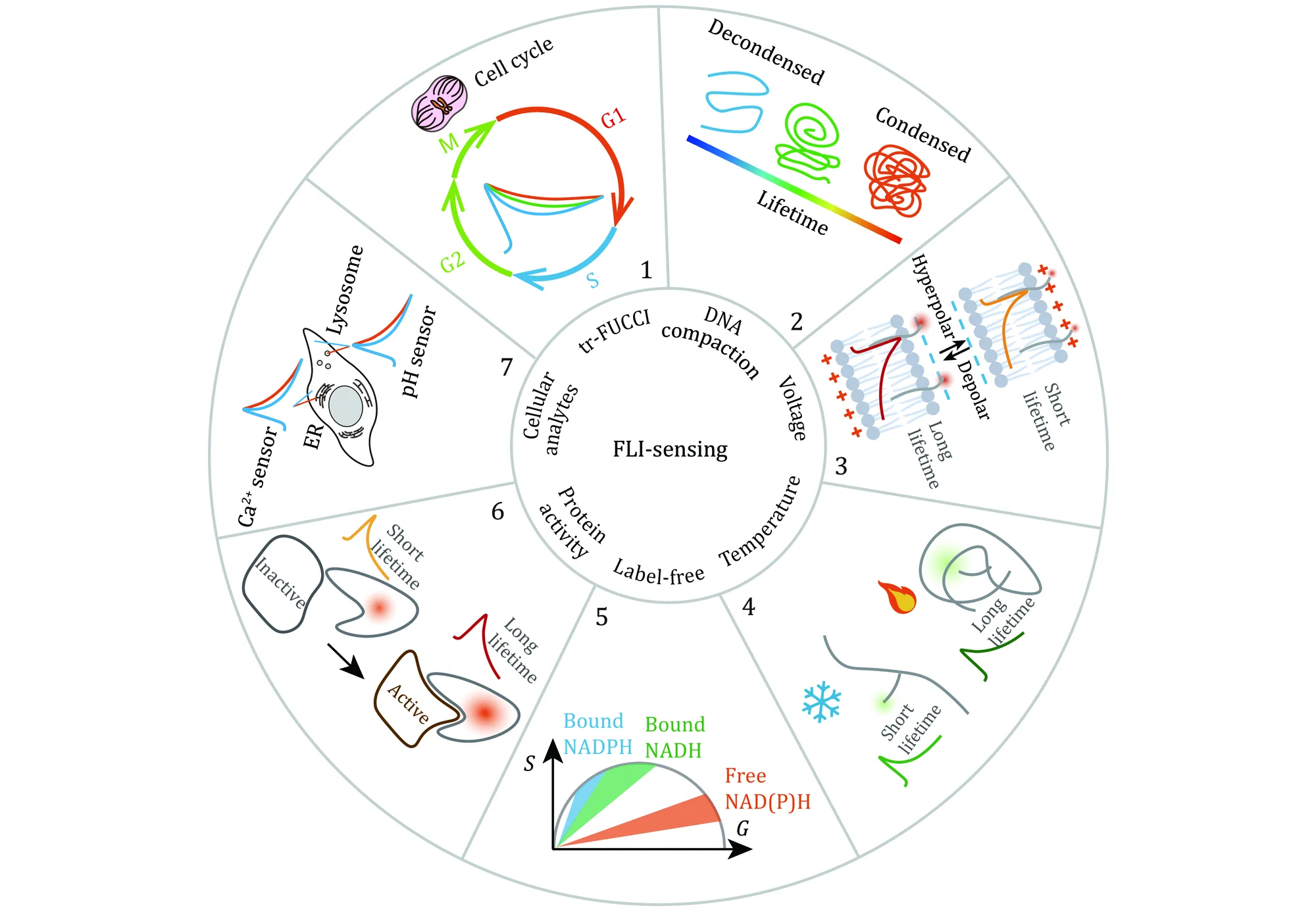
FLIM biosensors. Illustrative summary of environment‐sensing FLIM biosensors (excluding FLIM‐FRET). These biosensors monitor diverse cellular parameters in living cells, including: 1. cell cycle status (Tan *et al.* 2024); 2. molecular crowding (Levchenko *et al.* 2021); 3. membrane potential (van der Linden *et al.* 2021); 4. temperature (Okabe *et al.* 2012); 5. redox states (free versus protein‐bound NADH) (Sorrells *et al.* 2021); 6. protein activity (Mehl *et al.* 2024); 7. ion concentrations (*e*.*g*., Ca^2^^+^ (van der Linden *et al.* 2021) and H^+^ (Rennick *et al.* 2022))

FLIM is a robust tool to measure fluorescence resonance energy transfer (FRET) efficiency because of its independence from fluorophore intensity and the absence of bleed-through artifacts (Margineanu *et al.* 2016; Torrado *et al.* 2024). Consequently, FLIM-FRET has been successfully employed not only to detect protein conformational changes (Kagan *et al.* 2025), measure molecular distances (Cole *et al.* 2024), molecule concentration levels (Levitt *et al.* 2020; Sauer *et al.* 2014), assess binding kinetics, quantify protein–protein interactions (Kaufmann *et al.* 2020). In addition, the environmental sensitivity of fluorescence lifetimes has driven the development of sensors for various parameters, including pH (Bleeker *et al.* 2023; Goryashchenko *et al.* 2021; Herrera-Ochoa *et al.* 2022; Lazzari-Dean *et al.* 2022; Lin *et al.* 2003; Linders *et al.* 2022; Rennick *et al.* 2022), molecular crowding (Joron *et al.* 2023; Levchenko *et al.* 2021; Rieger *et al.* 2017), Ca^2+^ concentration (Celli *et al.* 2010; Simonyan *et al.* 2024; van der Linden *et al.* 2021, 2024; Zheng *et al.* 2018), Na^+^ concentration (Meyer *et al.* 2019; Schwarze *et al.* 2014), membrane voltage (Boggess *et al.* 2021; Brinks *et al.* 2015; Gest *et al.* 2021), and temperature (Inada *et al.* 2019; Liu *et al.* 2021; Okabe *et al.* 2012). Moreover, the intrinsic fluorescence of NADH — with distinct lifetimes in its free and protein-bound states — facilitates label-free FLIM applications (Lakowicz *et al.* 1992; Song *et al.* 2024; Sorrells *et al.* 2021; Stringari *et al.* 2012). The combination of FLIM with FUCCI enables cell cycle monitoring using a single spectral channel (Frei *et al.* 2022; Shirmanova *et al.* 2021; Tan *et al.* 2024). Figure 3 presents a selection of successful FLIM-based biosensors, highlighting their capacity to monitor dynamic cellular processes through these relative lifetime shifts. Additionally, two-photon imaging further extends FLIM’s utility to *in vivo* sensing (Kagan *et al.* 2025). For a concise overview of each sensor’s key design elements and performance characteristics, please see Table 2, which summarizes these features.

**Table 2:** A collection of representative FLI-biosensors

Category	Citation	Target	Readout mode	Sensor name	Sensing domain	Fluorophore	Chain type
Cell cycle	Frei *et al*. 2022	Cell cycle	FLIM	LT-Fucci(CA)	HaloTag9-hCdt1(1−100) and HaloTag7-hGem(1−110) degron fusions	MaP618-CA (rhodamine-CA)	Dual-construct
	Shirmanova *et al*. 2021	Cell cycle	FLIM	FUCCI-Red	hCdt1(30/120) & hGem(1/110) degradation motifs	mCherry & mKate2	Dual-construct
	Tan *et al*. 2024	Cell cycle	FLIM	tr-FUCCI	hGem(1−110) and hCdt1(1−100)Cy(-) degron fragments	mTurquoise2-T203G & mTurquoise2-T62C (circular-permuted CFP variants)	Dual-construct
Crowdness	Joron *et al*. 2023	Local densities within nuclear condensates	FLIM	mCherry	β-barrel fold	mCherry	Single-chain FP
	Levchenko *et al*. 2021	Genomic-DNA compaction (gene-rich vs. gene-poor domains)	FLIM	BrdU-incorporated DNA labeled with AlexaFluor 546	Bromodeoxyuridine (BrdU) moiety within DNA strands	AlexaFluor 546	n/a (chemical label incorporated into DNA)
	Levchenko *et al*. 2021	Genomic-DNA compaction (gene-rich vs. gene-poor domains)	FLIM (FRET)	Donor-acceptor labeled nucleotides incorporated into DNA	Halogenated nucleotides (CldU and IdU) incorporated into DNA	AlexaFluor 546 & 647	n/a (chemical label incorporated into DNA)
	Rieger *et al*. 2017	Respiratory supercomplex assembly	FLIM	CoxVIIIa-sEcGFP	sEcGFP	sEcGFP	Single-chain FP
Voltage	Brinks *et al*. 2015	Membrane voltage	FLIM	CAESR	Arch microbial rhodopsin domain	Citrine	Single-chain FP
	Boggess *et al*. 2021	Membrane potential in excitable cells	FLIM	VF2.1.Cl	Aniline donor-aromatic π system mediating PeT	Dichlorofluorescein scaffold	n/a (small-molecule)
Temperature	Okabe *et al*. 2012	Temperature	FLIM	FPT (fluorescent polymeric thermometer)	DBD-AA units (benzoxadiazole-based, water-sensitive fluorophore)	Benzoxadiazole derivative (DBD-AA)	n/a (synthetic polymeric probe)
	Liu *et al*. 2021	Temperature	FLIM	Core/shell NaGdF_4_:Er^3+^,Yb^3+^ / NaGdF_4_ upconverting nanoparticles	Er^3+^ and Yb^3+^ dopant energy levels within the NaGdF_4_ host lattice	Er^3+^ and Yb^3+^ dopants in NaGdF_4_ host lattice	n/a (inorganic nanoparticle probe, not a polypeptide)
NADH	Song *et al*. 2024	Endogenous NADH	FLIM	n/a	n/a	NADH autofluorescence	n/a
Protein activity	Mehl *et al*. 2024	Active conformation of endogenous Cdc42	FLIM	mero87-CBD	Cdc42-binding domain (CBD) activation reporter	Merocyanine derivative (“mero87”)	Protein−dye conjugate
Ca^2+^	van der Linden *et al*. 2021	Ca^2+^	FLIM	Tq-Ca-FLITS	Calmodulin/M13	mTurquoise2	Single-chain FP
	Simonyan *et al*. 2024	Ca^2+^	FLIM	GCaMP6s-BrUS and variants	Calmodulin/M13	cpBrUSLEE and variants	Single-chain FP
	van der Linden *et al*. 2024	Ca^2+^	FLIM	G-Ca-FLITS	Calmodulin/M13	mTurquoise2_T203Y	Single-chain FP
Na⁺	Meyer *et al*. 2019	Na⁺	FLIM	CoroNaGreen	Crown-ether	CoroNaGreen dye	n/a (small-molecule dye)
	Schwarze *et al*. 2014	Na⁺	FLIM	1,2,3-triazol-fluoroionophore 1	N-(o-methoxyphenyl)aza-15-crown-5 ionophore	7-Diethylaminocoumarin	n/a (small-molecule fluoroionophore)
pH	Goryashchenko *et al*. 2021	Extracellular pH	FLIM	SypHerExtra	SypHer3s (cpYFP‐based pH sensor)	GFP-based (SypHer3s derivative)	Single-chain FP
	Bleeker *et al*. 2023	pH	FLIM	Dedicated quinolinium-based probes	1-methyl-7-amino-quinolinium moiety	1-methyl-7-amino-quinolinium dye	n/a (small-molecule)
	Herrera-Ochoa *et al*. 2022	pH	FLIM	CdSe/ZnS-PH probe	D-penicillamine-histidine peptide	CdSe/ZnS quantum dot	n/a (nanoparticle)
	Lazzari-Dean *et al*. 2022	Lysosomal pH	FLIM	mScarlet-LAMP1 fusion protein	mScarlet	mScarlet	Single-chain FP
	Linders *et al*. 2022	Lysosomal pH	FLIM	LAMP1-RpHLuorin2	RpHLuorin2 (superecliptic pHluorin)	Ratiometric pHluorin2 (GFP variant)	Single-chain FP
	Rennick *et al*. 2022	Cell surface & endosomal pH	FLIM	TfR-mApple	mApple	mApple	Single-chain FP

Limitations: Despite its independence from probe concentration and immunity to photobleaching, FLIM-based biosensors typically exhibit limited dynamic range, with lifetime shifts of <500 ps under physiological conditions (Kagan *et al.* 2025; Tilden *et al.* 2024), making subtle biochemical changes hard to resolve against photon noise and autofluorescence backgrounds. Moreover, at photon budgets typical of live-cell FLIM (10^4^–10^5^ photons/pixel), lifetime precision is constrained to ∼100–300 ps — approaching many sensor Δ*τ* values — due to photon-statistical noise and instrument response (Ulku *et al.* 2020). In contrast, ratiometric intensity sensors often deliver >2–5-fold changes in emission ratio under similar conditions (Choe and Titov 2022), providing a substantially wider dynamic range and simpler quantification.

## INTEGRATION OF FLIM WITH OTHER SYSTEMS

### S-FLIM (Scipioni *et al.* 2021)

**Figure 4:**
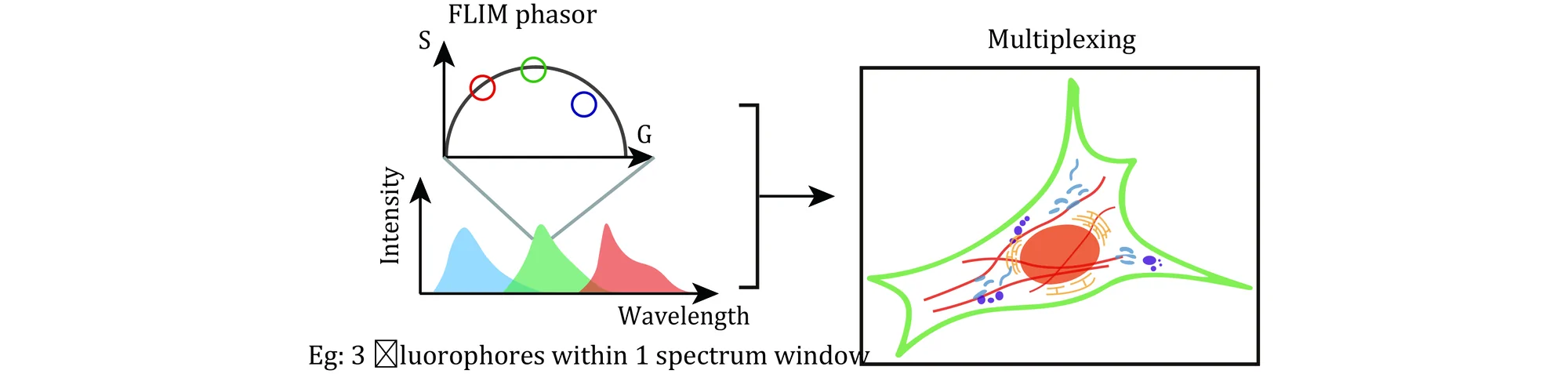
Multiplex imaging. General workflow for combining spectral separation and lifetime analysis to achieve multiplexed imaging. Multiple fluorophores are distinguished post‐acquisition based on both wavelength and lifetime

S-FLIM represents an innovative strategy that combines FLIM’s multiplexing capability with conventional multi-channel spectral information (Carlsson and Liljeborg 1997; Karpf *et al.* 2020; Wahl *et al.* 2020). By integrating these two dimensions, FLI‐S enhances the discrimination of fluorescent signals — even when they occupy the same spectral window — and significantly increases the number of targets that can be simultaneously imaged, particularly in customized multi-detector FLIM systems (Niehörster *et al.* 2016; Scipioni *et al.* 2021). Figure 4 provides a concise schematic of the multiplexing workflow that integrates spectral information with phasor-based FLIM analysis, enabling the simultaneous discrimination and imaging of multiple targets across the same and different spectral windows.

### FLI-SR

For time-domain FLIM, the laser-scanning method is naturally compatible with STED microscopy, making STED-FLIM a straightforward approach to super-resolution imaging (FLI-SR) (Auksorius *et al.* 2008; Bückers *et al.* 2011). While STED microscopy theoretically supports multicolor imaging through multiple depletion wavelengths (*e.g*., 592/595, 660, and 775 nm), in practice several issues constrain its utility. Dyes depleted with 592 or 660 nm continuous wave lasers bleach rapidly compared to those used with the pulsed 775 nm laser, and aligning multiple depletion beams precisely for colocalization is technically challenging — often leading to undesired cross-depletion of fluorochromes (Gonzalez Pisfil *et al.* 2022). Moreover, the heightened phototoxicity inherent in multicolor STED setups often restricts practical live-cell applications to two-color imaging (Zhang *et al.* 2023b). FLIM-STED, in theory, can achieve multiplexing of up to eight distinct targets by exploiting fluorescence lifetime differences, thereby overcoming some of the inherent limitations of conventional multicolor STED imaging (Bénard *et al.* 2024; Gonzalez Pisfil *et al.* 2022; Zhang *et al.* 2023b), and enhance its spatial resolution, enabling long-term imaging with reduced phototoxicity (Privitera *et al.* 2024; Tortarolo *et al.* 2019). Alternatively, fluorescence emission difference (FED) microscopy combined with FLIM offers a fundamentally different approach from STED-FLIM — enhancing spatial resolution without the need for high-intensity depletion beams, thereby substantially reducing photodamage compared to conventional STED-based methods (Wang *et al.* 2025).

### FLI-SMLM

FLI-SMLM has rapidly become a powerful modality for combining nanometer-scale localization with lifetime contrast, achieving sub-20 nm precision and per-molecule lifetime readouts in both wide-field (Oleksiievets *et al.* 2020) and confocal (Thiele *et al.* 2020) implementations (Datta *et al.* 2021; Oleksiievets *et al.* 2022a). Multiplexed strategies such as FL-PAINT exploit lifetime differences to image up to three targets simultaneously without fluid exchange (Oleksiievets *et al.* 2022b), while super-resolved smFRET and co-tracking in pMINFLUX combine sub-2 nm localization with lifetime measurements to reveal protein conformational dynamics at the nanometer scale (Cole *et al.* 2024; Masullo *et al.* 2021; Zähringer *et al.* 2023). pMINFLUX uses pulsed-interleaved excitation together with time-correlated single-photon counting to record both nanometer-precise positions and fluorescence lifetimes, overcoming conventional MINFLUX’s single-channel limitation by assigning photons to fluorophores based on their distinct lifetimes (Masullo *et al.* 2021). With this tool, FRET donor–acceptor pairs on DNA origami can be tracked with <2 nm precision across 4–100 nm distances, and has enabled dual-color co-tracking of two cell-surface receptors in live cells (Cole *et al.* 2024). Moreover, FLIM has also been combined with fluctuation-based super-resolution (Zeng *et al.* 2019) and image-scanning (ISM) techniques (Rossetta *et al.* 2022), providing viable routes to super-resolution FLIM using simpler, more accessible instrumentation.

### OUTLOOK

In fdFLIM, simultaneous decomposition of the multiple fluorescence lifetime components with a high temporal and spatial resolution remains challenging, since accurate decoupling requires significant light intensity change at multiple modulation frequencies (Lakowicz 2006). Further improvement is needed regarding reducing motion artifacts, improving photon efficiency, denoising, and resolving multi-exponential or non-exponential decays. Integrating fdFLIM with advanced deep learning techniques is expected to accelerate time-lapse imaging, allowing tracking of multiple organelles in live cells. Similarly, FLIM sensing applications are constrained by the relatively low fold-change in sensor response, which necessitates longer acquisition times and prevents imaging rates above 1 Hz (Zickus *et al.* 2020) — a performance level typically achieved in spectral imaging. This underscores the need for more sensitive biosensors and high-speed FLIM modalities.

Recent developments have also highlighted FLIM’s potential in areas such as autofluorescence reduction (Hwang *et al.* 2025), flow cytometry (Kanno *et al.* 2024; Karpf *et al.* 2020), cell sorting (Fahim *et al.* 2025), ultraplexing (Tan *et al.* 2024). As the complexity of high-dimensional FLIM data challenges traditional algorithms, deep learning approaches are increasingly being adopted, promising more efficient analysis and deeper insights into biological samples.

In terms of fluorophore development for multiplexing, while new photostable far-red and infrared probes have been engineered (Maiti *et al.* 2023; Matlashov *et al.* 2020), limitations still exist (Grimm and Lavis 2022). Continued advancements in brighter and more stable fluorophores will likely further enhance multiplexing capabilities, particularly for *in vivo* applications where longer wavelengths are essential.

Although early efforts have applied deep neural networks to accelerate FLIM fitting and perform basic denoising, most models still treat FLIM data as isolated 2D frames, overlooking its full multi-dimensional structure — comprising the *x* and *y* spatial axes, the decay-time histogram bins (*τ*), and additional *z*-stack or time-lapse (*t*) dimensions. By contrast, cutting-edge DL architectures — self-supervised spatial-redundancy transformers that denoise 3D + *t* fluorescence data with high SNR recovery (Li *et al.* 2023); deformable phase-space alignment TISR networks delivering >2× super-resolution and confidence-quantified time-lapse SR in live cells (Qiao *et al.* 2025); and deep learning — enabled digital spectral filtering for filter-free, multi-channel fluorescence microscopy (Dai *et al.* 2025) — have dramatically improved noise suppression, spatial resolution, and multiplexing capabilities across diverse fluorescence imaging modalities. To fully unlock FLIM’s potential, we need purpose-built networks that jointly ingest spatial coordinates, decay kinetics, and temporal context, integrate instrument-response modeling or phasor-domain constraints, and leverage physics-informed or self-supervised pretraining on large unlabeled FLIM datasets. Such 4D or even 5D DL frameworks promise to overcome today’s bottlenecks — low acquisition speed and photon-limited noise — delivering real-time, high-SNR lifetime maps and opening FLIM to dynamic, live-cell applications beyond current limits.

## References

[bAdhikari2023] (2023). Review of fluorescence lifetime imaging microscopy (FLIM) data analysis using machine learning. J Exp Theor Anal.

[bAoyama2023] Aoyama T, Sugimoto N, Sato Y (2023) Application of fluorescence lifetimes to multi-imaging analysis in plant cells. bioRxiv. https://doi.org/10.1101/2023.08.28.555227

[bAuksorius2008] (2008). Stimulated emission depletion microscopy with a supercontinuum source and fluorescence lifetime imaging. Opt Lett.

[bBacia2006] (2006). Fluorescence cross-correlation spectroscopy in living cells. Nat Methods.

[bBailey1953] (1953). The determination of the fluorescence lifetimes of dissolved substances by a phase shift method. J Chem Phys.

[bBecker2012] Becker W (2012) Fluorescence lifetime imaging--techniques and applications. J Microsc 247(2): 119–136

[bBecker2001] Becker W, Benndorf K, Bergmann A, Biskup C, Koenig K, Tirlapur U, Zimmer T (2001) FRET measurements by TCSPC laser scanning microscopy. Proc SPIE 4431, Photon Migration, Optical Coherence Tomography, and Microscopy. https://doi.org/10.1117/12.447406

[bBnard2024] (2024). Combining sophisticated fast FLIM, confocal microscopy, and STED nanoscopy for live-cell imaging of tunneling nanotubes. Life Sci Alliance.

[bBerezin2010] (2010). Fluorescence lifetime measurements and biological imaging. Chem Rev.

[bBernardi2023] (2023). Phasor identifier: a cloud-based analysis of phasor-FLIM data on Python notebooks. Biophys Rep (N Y).

[bBindels2017] (2017). mScarlet: a bright monomeric red fluorescent protein for cellular imaging. Nat Methods.

[bBlacker2014] (2014). Separating NADH and NADPH fluorescence in live cells and tissues using FLIM. Nat Commun.

[bBleeker2023] (2023). Quinolinium-based fluorescent probes for dynamic pH monitoring in aqueous media at high pH using fluorescence lifetime imaging. ACS Sens.

[bBoens2007] (2007). Fluorescence lifetime standards for time and frequency domain fluorescence spectroscopy. Anal Chem.

[bBogdanova2024] (2024). Fluorescence lifetime multiplexing with fluorogen activating protein FAST variants. Commun Biol.

[bBoggess2021] (2021). Fluorescence lifetime predicts performance of voltage sensitive fluorophores in cardiomyocytes and neurons. RSC Chem Biol.

[bBowman2023] (2023). Wide-field fluorescence lifetime imaging of neuron spiking and subthreshold activity *in vivo*. Science.

[bBright1990] (1990). Advances in multifrequency phase and modulation fluorescence analysis. Crit Rev Anal Chem.

[bBrinks2015] (2015). Two-photon lifetime imaging of voltage indicating proteins as a probe of absolute membrane voltage. Biophys J.

[bBcherl2010] Bücherl C, Aker J, de Vries S, Borst JW (2010) Probing protein-protein interactions with FRET-FLIM. In: Hennig L, Köhler C (eds.) Plant developmental biology: methods and protocols. Totowa: Humana Press, pp 389–399

[bBckers2011] (2011). Simultaneous multi-lifetime multi-color STED imaging for colocalization analyses. Opt Express.

[bBugiel1989] (1989). Investigations of cells by fluorescence laser scanning microscopy with subnanosecond resolution. Lasers Life Sci.

[bCalleja2003] Calleja V, Ameer-Beg SM, Vojnovic B, Woscholski R, Downward J, Larijani B (2003) Monitoring conformational changes of proteins in cells by fluorescence lifetime imaging microscopy. Biochem J 372(Pt 1): 33–40

[bCanty2018] (2018). Peak emission wavelength and fluorescence lifetime are coupled in far-red, GFP-like fluorescent proteins. PLoS One.

[bCarlsson1997] (1997). Confocal fluorescence microscopy using spectral and lifetime information to simultaneously record four fluorophores with high channel separation. J Microsc.

[bCelli2010] (2010). The epidermal Ca^2+^ gradient: measurement using the phasor representation of fluorescent lifetime imaging. Biophys J.

[bChoe2022] (2022). Genetically encoded tools for measuring and manipulating metabolism. Nat Chem Biol.

[bCole2024] (2024). Super-resolved FRET and co-tracking in pMINFLUX. Nat Photon,.

[bDai2025] (2025). Deep learning-enabled filter-free fluorescence microscope. Sci Adv.

[bDatta2021] (2021). Recent innovations in fluorescence lifetime imaging microscopy for biology and medicine. J Biomed Opt.

[bDigman2008] (2008). The phasor approach to fluorescence lifetime imaging analysis. Biophys J.

[bDysli2017] (2017). Fluorescence lifetime imaging ophthalmoscopy. Prog Retin Eye Res.

[bEggan2024] (2024). Ligand-coupled conformational changes in a cyclic nucleotide-gated ion channel revealed by time-resolved transition metal ion FRET. Elife,.

[bEl2024] (2024). Multiplexed *in vivo* imaging with fluorescence lifetime-modulating tags. Adv Sci.

[bElder2006] (2006). The application of frequency-domain fluorescence lifetime imaging microscopy as a quantitative analytical tool for microfluidic devices. Opt Express.

[bFahim2025] (2025). Fluorescence lifetime sorting reveals tunable enzyme interactions within cytoplasmic condensates. J Cell Biol.

[bFrei2022] (2022). Live-cell fluorescence lifetime multiplexing using synthetic fluorescent probes. ACS Chem Biol.

[bGadella2023] (2023). mScarlet3: a brilliant and fast-maturing red fluorescent protein. Nat Methods.

[bGao2020] (2020). FLIMJ: an open-source ImageJ toolkit for fluorescence lifetime image data analysis. PLoS One.

[bGest2021] (2021). VoltageFluor dyes and fluorescence lifetime imaging for optical measurement of membrane potential. Method Enzymol.

[bGonzalez2022] (2022). Stimulated emission depletion microscopy with a single depletion laser using five fluorochromes and fluorescence lifetime phasor separation. Sci Rep.

[bGoryashchenko2021] (2021). FLIM-based intracellular and extracellular pH measurements using genetically encoded pH sensor. Biosensors.

[b43] (2023). FLUTE: a Python GUI for interactive phasor analysis of FLIM data. Biol Imaging.

[bGrimm2022] (2022). Caveat fluorophore: an insiders' guide to small-molecule fluorescent labels. Nat Methods.

[bHeliot2021] (2021). Simple phasor-based deep neural network for fluorescence lifetime imaging microscopy. Sci Rep.

[bHerreraOchoa2022] (2022). A novel quantum dot-based pH probe for long-term fluorescence lifetime imaging microscopy experiments in living cells. ACS Appl Mater Interfaces.

[bHwang2025] (2025). A robust method for autofluorescence-free immunofluorescence using high-speed fluorescence lifetime imaging microscopy. Sci Rep.

[bInada2019] (2019). Temperature imaging using a cationic linear fluorescent polymeric thermometer and fluorescence lifetime imaging microscopy. Nat Protoc.

[bJaresErijman2003] (2003). FRET imaging. Nat Biotechnol.

[bJoron2023] (2023). Fluorescent protein lifetimes report densities and phases of nuclear condensates during embryonic stem-cell differentiation. Nat Commun.

[bKagan2025] (2025). Genetically encoded biosensor for fluorescence lifetime imaging of PTEN dynamics in the intact brain. Nat Methods.

[bKanno2024] (2024). High-throughput fluorescence lifetime imaging flow cytometry. Nat Commun.

[bKapitany2024] (2024). Single-sample image-fusion upsampling of fluorescence lifetime images. Sci Adv.

[bKapsiani2025] Kapsiani S, Läubli NF, Ward EN, Fernandez-Villegas A, Mazumder B, Kaminski CF, Schierle GSK (2025) Deep learning for fluorescence lifetime predictions enables high-throughput in vivo imaging. bioRxiv. https://doi.org/10.1101/2025.02.20.639036

[bKarpf2020] (2020). Spectro-temporal encoded multiphoton microscopy and fluorescence lifetime imaging at kilohertz frame-rates. Nat Commun.

[bKaufmann2020] (2020). Direct measurement of protein-protein interactions by FLIM-FRET at UV laser-induced DNA damage sites in living cells. Nucleic Acids Res.

[bKlarenbeek2015] (2015). Fourth-generation Epac-based FRET sensors for cAMP feature exceptional brightness, photostability and dynamic range: characterization of dedicated sensors for FLIM, for ratiometry and with high affinity. PLoS One.

[bKoenig1994] Koenig K, Schneckenburger H (1994) Laser-induced dental caries and plaque diagnosis on patients by sensitive autofluorescence spectroscopy and time-gated video imaging: preliminary studies. Proc SPIE 2128, Laser Surgery: Advanced Characterization, Therapeutics, and Systems IV. https://doi.org/10.1117/12.184924

[bKnig2018] König K (2018) 1 Brief history of fluorescence lifetime imaging. In: König K (ed.) Multiphoton microscopy and fluorescence lifetime imaging: applications in biology and medicine. Berlin: De Gruyter, pp 3–16

[bKnig1999] (1999). Time-gated *in vivo* autofluorescence imaging of dental caries. Cell Mol Biol.

[bLakowicz2006] Lakowicz JR (2006) Introduction to fluorescence. In: Lakowicz JR (ed.) Principles of fluorescence spectroscopy. Boston: Springer, pp 1–26

[bLakowicz1992] (1992). Fluorescence lifetime imaging of free and protein-bound NADH. Proc Natl Acad Sci USA.

[bLazzariDean2022] Lazzari-Dean JR, Ingaramo MC, Wang JCK, Yong J, Ingaramo M (2022) mScarlet fluorescence lifetime reports lysosomal pH quantitatively. https://doi.org/10.5281/zenodo.6363342

[bLevchenko2021] (2021). Fluorescence lifetime imaging for studying DNA compaction and gene activities. Light Sci Appl.

[bLevitt2020] (2020). Quantitative real-time imaging of intracellular FRET biosensor dynamics using rapid multi-beam confocal FLIM. Sci Rep.

[bLi2023] (2023). Spatial redundancy transformer for self-supervised fluorescence image denoising. Nat Comput Sci.

[bLi2025] (2025). *In vivo* neurodynamics mapping via high-speed two-photon fluorescence lifetime volumetric projection microscopy. Adv Sci.

[bLin2025] (2025). UNET-FLIM: a deep learning-based lifetime determination method facilitating real-time monitoring of rapid lysosomal pH variations in living cells. Anal Chem.

[bLin2003] Lin H-J, Herman P, Lakowicz JR (2003) Fluorescence lifetime-resolved pH imaging of living cells. Cytom Part A 52A(2): 77–89

[bLinders2022] (2022). Fluorescence lifetime imaging of pH along the secretory pathway. ACS Chem Biol.

[bLippincottSchwartz2003] (2003). Development and use of fluorescent protein markers in living cells. Science.

[bLiu2021] (2021). Fast wide-field upconversion luminescence lifetime thermometry enabled by single-shot compressed ultrahigh-speed imaging. Nat Commun.

[bMaiti2023] (2023). Structural and photophysical characterization of the small ultra-red fluorescent protein. Nat Commun.

[bMamontova2018] (2018). Bright GFP with subnanosecond fluorescence lifetime. Sci Rep.

[bManna2018] (2018). Directed evolution of excited state lifetime and brightness in FusionRed using a microfluidic sorter. Integr Biol.

[bMannam2020] (2020). Machine learning for faster and smarter fluorescence lifetime imaging microscopy. J Phys Photonics.

[bMargineanu2016] (2016). Screening for protein-protein interactions using Förster resonance energy transfer (FRET) and fluorescence lifetime imaging microscopy (FLIM). Sci Rep.

[bMasullo2021] (2021). Pulsed interleaved MINFLUX. Nano Lett.

[bMatlashov2020] (2020). A set of monomeric near-infrared fluorescent proteins for multicolor imaging across scales. Nat Commun.

[bMehl2024] (2024). Live-cell biosensors based on the fluorescence lifetime of environment-sensing dyes. Cell Rep Methods.

[bMeyer2019] (2019). Quantitative determination of cellular [Na^+^] by fluorescence lifetime imaging with CoroNaGreen. J Gen Physiol.

[bMukherjee2024] (2024). "Radical" differences between two FLIM microscopes affect interpretation of cell signaling dynamics. iScience.

[bMukherjee2022] (2022). Directed evolution of a bright variant of mCherry: suppression of nonradiative decay by fluorescence lifetime selections. J Phys Chem B.

[bNiehrster2016] (2016). Multi-target spectrally resolved fluorescence lifetime imaging microscopy. Nat Methods.

[bOkabe2012] (2012). Intracellular temperature mapping with a fluorescent polymeric thermometer and fluorescence lifetime imaging microscopy. Nat Commun.

[bOleksiievets2022a] (2022a). Single-molecule fluorescence lifetime imaging using wide-field and confocal-laser scanning microscopy: a comparative analysis. Nano Lett.

[bOleksiievets2022b] (2022b). Fluorescence lifetime DNA-PAINT for multiplexed super-resolution imaging of cells. Commun Biol.

[bOleksiievets2020] (2020). Wide-field fluorescence lifetime imaging of single molecules. J Phys Chem A.

[bPelet2004] (2004). A fast global fitting algorithm for fluorescence lifetime imaging microscopy based on image segmentation. Biophys J.

[bPrivitera2024] (2024). Super-resolved analysis of colocalization between replication and transcription along the cell cycle in a model of oncogene activation. Commun Biol.

[bQiao2022] Qiao C (2022) BioSR+: dataset extension of biological images for super-resolution microscopy. https://zenodo.org/records/7115540

[bQiao2021] (2021). Evaluation and development of deep neural networks for image super-resolution in optical microscopy. Nat Methods.

[bQiao2025] Qiao C, Liu S, Wang Y, Xu W, Geng X, Jiang T, Zhang J, Meng Q, Qiao H, Li D, Dai Q (2025) A neural network for long-term super-resolution imaging of live cells with reliable confidence quantification. Nat Biotechnol. https://doi.org/10.1038/s41587-025-02553-8

[bRaspe2016] (2016). siFLIM: single-image frequency-domain FLIM provides fast and photon-efficient lifetime data. Nat Methods.

[bRedford2005] (2005). Polar plot representation for frequency-domain analysis of fluorescence lifetimes. J Fluoresc.

[bRennick2022] (2022). Resolving subcellular pH with a quantitative fluorescent lifetime biosensor. Nat Commun.

[bRieger2017] (2017). Lifetime imaging of GFP at CoxVIIIa reports respiratory supercomplex assembly in live cells. Sci Rep.

[bRossetta2022] (2022). The BrightEyes-TTM as an open-source time-tagging module for democratising single-photon microscopy. Nat Commun.

[bSauer2014] (2014). Confocal FLIM of genetically encoded FRET sensors for quantitative Ca^2+^ imaging. Cold Spring Harb Protoc.

[bSchindelin2012] (2012). Fiji: an open-source platform for biological-image analysis. Nat Methods.

[bSchneckenburger1985] (1985). Time-resolved microfluorescence in biomedical diagnosis. Opt Engin.

[bSchneckenburger1987] (1987). Picosecond fluorescence microscopy for measuring chlorophyll and porphyrin components in conifers and cultured cells. Lasers Life Sci.

[bSchrimpf2018] (2018). PAM: a framework for integrated analysis of imaging, single-molecule, and ensemble fluorescence data. Biophys J.

[bSchwarze2014] (2014). Fluorescence lifetime-based sensing of sodium by an optode. Chem Commun.

[bScipioni2021] (2021). Phasor S-FLIM: a new paradigm for fast and robust spectral fluorescence lifetime imaging. Nat Methods.

[bShen2024] (2024). Overcoming photon and spatiotemporal sparsity in fluorescence lifetime imaging with SparseFLIM. Commun Biol.

[bShirmanova2021] (2021). FUCCI-Red: a single-color cell cycle indicator for fluorescence lifetime imaging. Cell Mol Life Sci.

[bSimonyan2024] (2024). Calcium indicators with fluorescence lifetime-based signal readout: a structure-function study. Int J Mol Sci.

[bSmith2019] (2019). Fast fit-free analysis of fluorescence lifetime imaging via deep learning. Proc Natl Acad Sci USA.

[bSong2024] (2024). Visualizing subcellular changes in the NAD(H) pool size versus redox state using fluorescence lifetime imaging microscopy of NADH. Commun Biol.

[bSorrells2021] (2021). Label-free characterization of single extracellular vesicles using two-photon fluorescence lifetime imaging microscopy of NAD(P)H. Sci Rep.

[bStarling2023] (2023). Multicolor lifetime imaging and its application to HIV-1 uptake. Nat Commun.

[bStringari2012] (2012). Metabolic trajectory of cellular differentiation in small intestine by Phasor fluorescence lifetime microscopy of NADH. Sci Rep.

[bTan2024] Tan Z, Hsiung C-H H, Feng J, Zhang Y, Chen J, Sun K, Lu P, Zang J, Yang W, Gao Y, Yin J, Zhu T, Ye Y, Wan Y, Zhang X (2024) Time-resolved fluorescent proteins towards fluorescence microscopy in the temporal and spectral domains. bioRxiv. https://doi.org/10.1101/2024.12.16.628778

[bThiele2020] (2020). Confocal fluorescence-lifetime single-molecule localization microscopy. ACS Nano.

[bTilden2024] (2024). A Cre-dependent reporter mouse for quantitative real-time imaging of protein kinase A activity dynamics. Sci Rep.

[bTorrado2024] (2024). Fluorescence lifetime imaging microscopy. Nat Rev Methods Primers.

[bTortarolo2019] (2019). Photon-separation to enhance the spatial resolution of pulsed STED microscopy. Nanoscale.

[bUlku2020] (2020). Wide-field time-gated SPAD imager for phasor-based FLIM applications. Methods Appl Fluoresc.

[bVallmitjana2020] (2020). Blind resolution of lifetime components in individual pixels of fluorescence lifetime images using the phasor approach. J Phys Chem B.

[bvan2021] (2021). A turquoise fluorescence lifetime-based biosensor for quantitative imaging of intracellular calcium. Nat Commun.

[bvan2024] van der Linden FH, Thornquist SC, ter Beek RM, Huijts JY, Hink MA, Gadella Jr TWJ, Maimon G, Goedhart J (2024) A green lifetime biosensor for calcium that remains bright over its full dynamic range. bioRxiv. https://doi.org/10.1101/2024.11.05.622032

[bWahl2020] (2020). Photon arrival time tagging with many channels, sub-nanosecond deadtime, very high throughput, and fiber optic remote synchronization. Rev Sci Instrum.

[bWang2025] (2025). Phasor-FSTM: a new paradigm for multicolor super-resolution imaging of living cells based on fluorescence modulation and lifetime multiplexing. Light Sci Appl.

[bWetzker2025] Wetzker C, Zoccoler ML, Iarovenko S, Okafornta CW, Nobst A, Hartmann H, Müller-Reichert T, Haase R, Fabig G (2025) Fluorescence lifetime unmixing: a new workflow for FLIM live-cell imaging. bioRxiv. https://doi.org/10.1101/2025.03.05.641717

[bXiao2023] (2023). Deep learning enhanced fast fluorescence lifetime imaging with a few photons. Optica.

[bYao2019] (2019). Net-FLICS: fast quantitative wide-field fluorescence lifetime imaging with compressed sensing – a deep learning approach. Light Sci Appl.

[bZhringer2023] (2023). Combining pMINFLUX, graphene energy transfer and DNA-PAINT for nanometer precise 3D super-resolution microscopy. Light Sci Appl.

[bZeng2019] (2019). Lifetime super-resolution optical fluctuation imaging. J Microsc.

[bZhang2023a] (2023a). Quantitative assessment of near-infrared fluorescent proteins. Nat Methods.

[bZhang2023b] Zhang Z, Huang Y, Tao W, Wei Y, Xu L, Gong W, Zhang Y, Zhou J, Cao L, Liu Y, Han Y, Kuang C, Liu X (2023b) Multi-color live-cell optical nanoscopy using phasor analysis. bioRxiv. https://doi.org/10.1101/2023.08.04.551988

[bZheng2018] (2018). Monitoring intracellular nanomolar calcium using fluorescence lifetime imaging. Nat Protoc.

[bZickus2020] (2020). Fluorescence lifetime imaging with a megapixel SPAD camera and neural network lifetime estimation. Sci Rep.

